# Supplementary material for: Learning curve and surgical outcome of robotic assisted colorectal surgery with ERAS program
Source: Sci Rep. 2022 Nov 29;12:20566. doi: 10.1038/s41598-022-24665-w (PMC9709162; doi:10.1038/s41598-022-24665-w)
Supplement: Supplementary file 1 — Supplementary Information 1. [file 41598_2022_24665_MOESM1_ESM.zip › LSC Raw data-Ñ[▒K/107/H_ñw╝╨░O▒Kñσ.pdf]

正式摘要(電子簽章：0000002052236888)

W52 / W52 ☐ 英文標題(注意:無英文姓名-請補病人基本檔!) ☐ 【讀取】憑證

臺中榮民總醫院

出院病歷摘要

轉入號：　　　　轉出號：

[redacted]

(8)流水編號	
(9)入院日期	109/12/02 (2020) 直腸外科 W52 - 171
(10)轉科日期	
(11)出院日期	109/12/14 (2020) 住院天數計 12 日

診斷

- 入院 :Sigmoid colon cancer
  Type 2 DM, hyperuriccemia, hypercholesterolemia
- 出院 :Sigmoid colon cancer s/p laparoscopic anterior resection on 2020/12/4
  Type 2 DM, hyperuriccemia, hypercholesterolemia

主訴

- bloody stool for about 2 months

病史

- This 84 years old male patient has past history of type 2 DM, hyperuriccemia, hypercholesterolemia. He recieved appendectomy several decades ago and TURP for BPH on 2020/5. He complained changed bowel habbit this year and the symptom progressed to bloody stool in recent two months. He came to 光田 for help initially and colonoscopy with biopsy showed malignant tumor in sigmoid colon. Thus, he was transferred to our hospital for help and was admitted to ward for surgical treatment.

體檢發現

-
  - General appearance :
    - easy looking , conscious clear , without cardiopulmonary distress
    - Vital signs自動帶入date 體溫(BT) 脈搏(P) 呼吸(R) 血壓(BP) 身高(BH) 體重(BW) BMI
      20201202 36.6℃ 82次/分 19次/分 128/89mmHg 161.5cm 78.7kg 30.2
  - HEENT :
    - Eyes: no anemic conjunctiva, no icteric sclera, normal EOM
    - ENT: no otorrhea, no rhinorrhea, no erythematous mucosa, no injected tonsil, no deformity
  - Neck: supple, freely movable, trachea midline,no deformity, no engorged jugular vein , no palpable thyroid or lymph node enlargement
  - Chest:
    - Inspection: Grossly normal, symmetric and fully expansion, no accessory muscle used, no operation scar, no spider angiomata, no gynecomastia
    - Palpation: no palpable mass or nodule, no subcutaneous emphysema, no tenderness
    - Ausculation: clear breathing sound
  - Heart:
    - Percussion: normal heart size
    - Ausculation: regular heart beat, normal S1, S2, no gallop, no murmur
  - Abdomen:
    - Inspection: in
      - flat contour, symetrically, no scar, no stria, no dilated veins, no rashes
    - Ausculation: normoactive bowel sound, no abdominal bruits
    - palpation: soft, no muscle guarding or rigidity, no local tenderness, no rebounding pain, no palpable mass, no palpable pulsation
    - Percussion: no shifting dulllness, no hepatomegaly, no splenomagaly
  -

Peripheral pulsation :	CA	BA	RA	FA	PA	PTA	DPA
Right side :	++	++	++	++	++	++	++/+++
Left side :	++	++	++	++	++	++	++/+++

  - Genito-anus: no tenderness or palpable mass lesion or rectal shelf, normal external genitalia, normal anal tone, brown stool

CRS CONDITION

  - CEA: 2.72 ng/ml
  -
    - Lung

手術日期及方法

- 手術日期及方法:20201204 1. Laparoscopic anterior resection with end-to-end anastomosis, endo-GIA 60/4.1mm*1, ILS 29mm, re-inforcement with silk: nil, bilateral doughnuts: intact, air leak test: done, no air leak.
  2. High ligation (+): IMA above left colic artery.
  3. Cut margin: proximal 7cm, distal 5cm.
  4. Reperitonealization: nil.
  5. Take down of splenic flexure: nil.
  6. One 12Fr. Penrose drain at pelvis.
  7. Intraoperative colonoscopy was done.

住院治療經過

- After admission, abdominal CT was arranged and the result showed suspected sigmoid colon cancer that invaded to non-peritonealized pericolonic or perirectal tissues without distant metastasis. Thus, operation with laparoscopic anterior resection was done on 2020/12/4 and endotracheal tube was removed at POR. In ward, fever was noted so culture was collected. we let him try water on POD3 and Bland I diet on POD6. Dyspnea improved. Under the stable condition, he was discharged on 12/14.

合併症

- nil

一般檢查

- 累積報告 :

DATE	TIME	ALB	TP	BIL.T	BIL.D	ALKP	AST	ALT	LDH	CHOL	TG	HDL-C	LDL	UA	NH3	AMY
1091202	1716	4.0														
1091205	0105	3.5		0.42	0.13	64	12	10								
1091207	0940						13	7								
1091210	0927	2.7		0.38	0.18			9								

DATE	TIME	WBC	RBC	HGB	HCT	MCV	PLT	BLST	NEUT	BAND	SEG	LYM	MON	EOS	BAS
1091202	1716	8020	4.53	12.3	38.6	85.2									
1091205	0104	10620		12.0	37.5		224		83.2			11.8	3.6	1.1	0.3
1091207	0945	10520		11.8	35.6		202		80.5			11.9	6.7	0.6	0.3
1091210	0931	8550	3.71	10.7	31.8	85.7	217		74.1			13.6	7.7	4.2	0.4
1091214	0853	7660		10.7	32.9		402		70.0			17.4	5.4	6.7	0.5

DATE	TIME	NA	K	CL	CA	BUN	CREAT	Mg	P	CRP	ESR	PCT	LIPASE	LACTATE
1091202	1716	140	3.8	103	9.4	21	1.03							
1091205	0105	140	3.4		8.8	15	0.97							
1091207	0940	138	3.6			9	1.05							
1091210	0927	134	3.5	94	8.5	14	1.01							
1091214	0956	136	3.6			12	0.91							

DATE	TIME	GLUCOSE	PAB	TRF	ZN	CU	KETONE	CK	CKMB	PT-P	PT-C	APTT-P	APTT-C	TRO-T

1091202	1716	117												

DATE	TIME	SP-GR	PH	PROT	GLUC	KETON	BILI	UROB	OB	NITR	LEU	RBC	WBC	EPCEL
1091207	1524	1.016	5.5	1+	250.0	Negative	Negative	0.2	Trace	Negative	Negative	0-2	0-2	0-1

- 放射線報告 :檢驗項目 **:SHOULDER(BILAT)** 報告日期 **:1091210**

主 訴：
Result:
Finding:
Shoulder, right:
> OA (osteoarthritis) change with marginal spurs formation and uneven articular surface in the acromioclavicular (AC) joints.
> Presence of subacromial spur, hyperostosis at greater humeral tuberosity and marked acromiohumeral space narrowing of bilateral shoulders, R/O shoulder impingement with rotator cuff tear.
> Old fracture of left ribs
Impression:
As above description.

檢驗項目 **:CHEST AP VIEW (PORTABLE)** 報告日期 **:1091210**
主 訴：
Result:
Finding:
> Enlarged cardiac shadow
> Tortuosity and calcifications of thoracic aorta
> Enlargement of bilateral hila
> Hazy opacities with increased infiltration in bilateral lower lungs
> Blunting of bilateral CP angles, R/O pleural effusion or chronic pleural change
> Thoracolumbar spine is hard to evaluate in this study
> Old fractures through left ribs
Impression:
As above description.

檢驗項目 **:CHEST PA VIEW** 報告日期 **:1091210**
主 訴：
Result:
Finding:
> Enlarged cardiac shadow
> Tortuosity and calcifications of thoracic aorta
> Enlargement of bilateral hila
> Hazy opacities with increased infiltration in bilateral lungs
> Bilateral costophrenic angle blunting, maybe due to pleural effusion or pleural disease.
> Degenerative changes with osteophytes of the thoracolumbar spine
> Old fractures through left ribs
Impression:
As above description.

檢驗項目 **:CT ABDOMEN-SURVEY** 報告日期 **:1091207**
主 訴：
Result:
Finding:
Imaging Report Form for Colorectal Carcinoma (AJCC 8th)
1. Imaging Date: 20201203
Imaging Modality, Imaging by
CT scan
Imaging Protocol: CT scan of abdomen with IV and oral contrast enhancement:
2. Tumor location / Size
Location:
Sigmoid
Size:
Measurable: at least 6.4 cm (largest diameter)
3. Tumor invasion
Tumor invades non-peritonealized pericolonic or perirectal tissues
4. Regional nodal metastasis
No
5. Distant metastasis (In this study)
No or Equivocal
6. Other findings: > Focal asymmetrical wall thickening at least 6.4cm in length over S-colon (Axi#60-#65, Cor#20-#27) with stranding of pericolic fat plane & small pericolic lymph nodes, less than 1cm in size, R/I colon cancer.
> Multiple tiny gallbladder stones
> Multiple smaller than 2.2 cm renal cysts over bilateral kidneys
> A 2.4 cm juxtapapillary diverticulum arising from 2nd portion of duodenum is noted(Axi#31, Cor#23).
> Defect is found in base of U-bladder may be due to S/P TURP.
> mild fatty liver
> spleen, pancreas and bilateral adrenal glands appear not unusual.
> No significant enlarged lymph node in retroperitoneum or bilateral inguinal regions can be noted
> Slight subpleural infiltration of bil. lower lungs, in favor of chronic inflammatory process.
> No obvious bony destruction, except DJD changes.
Impression:
1. R/I S-colon cancer. Please correlate with colonoscopy.
2. For other details, please see the above descriptions.

特殊檢查

病理報告

- 活體組織切片病理報告 手術日期：**109/12/05** 病理號：**S0939631**

診斷醫師:文芙卿 報告日期:109/12/11
Pathologic diagnosis:
1. Sigmoid colon, laparoscopic anterior resection —
Adenocarcinoma, moderately differentiated.
Tumor invades through muscularis propria into mesocolic soft tissue.
The bilateral cut ends and mesocolic lymph nodes (0/22) are not involved.
2. IMA root, dissection — Unremarkable. Negative for metastatic carcinoma (0/4).
3. Colon, cut end, distal, resection — Unremarkable.
Ancillary study for diagnosis:
1. IHC stains for PMS2 and MSH6 are done on section F. Both markers are
preserved.
2. VVG stain done on section F, no venous invasion is highlighted.
Prognostic and predictive factor:
1. Tumor size: 8.5x7 cm.
2. Depth of invasion: Pericolorectal tissue.
3. Histologic grade: G2: Moderately differentiated.
4. Angiolymphatic invasion: Not identified.
5. Perineural invasion: Not identified.
6. Tumor deposits (discontinuous extramural extension): Not identified.
7. Macroscopic tumor perforation: Not identified.

- 8. Surgical Margins: a) Proximal Margin: Uninvolved by invasive carcinoma, distance of tumor from margin: 7 cm. b) Distal Margin: Uninvolved by invasive carcinoma, distance of tumor from margin: 2 cm. c) Radial or Mesenteric Margin: Uninvolved by invasive carcinoma, distance of tumor from margin: more than 1 cm.
  9. Lymph node status: Uninvolved (0/26).
  10. Extranodal involvement: Not identified.
  11. Tumor regression grading S/P CCRT: No known presurgical therapy.
  12. Type of polyp in which invasive carcinoma arose: Not identified.
  13. Additional pathologic findings: Not identified.
  14. Pathological TNM Stage: pT3N0(According to the eighth edition, American Joint Committee on Cancer Staging Guidelines for Tumors).
  15. TNM descriptors: Not applicable.
  Gross description:
  The specimen consists of 1) a segment of colon, 16 cm in length and 6 cm in proximal cut end circumference, 9.5 cm in distal circumference, and mesocolic soft tissue. A gray tan firm annular elevated and centrally ulcerative tumor, 8.5x7 cm, is noted, 2 cm to the nearest cut end. On cross section, the tumor invades to the mesocolic soft tissue with more than 10 mm to the radial margin. The both cut ends are free of tumor grossly. 2) IMA root, a piece of tan yellow soft tissue, 5.5x3.5x1 cm. 3) distal cut end, a piece of tan mucosa-covered soft tissue, 2x2x1 cm. Representative part for section: A) specimen 2 B) specimen 3 all for section C) distal cut end D) proximal cut end E-G) tumor and serosa H) tumor and CFM J-K) tumor L) tumor and non-tumor part M-P) lymph nodes. C-F) tumor G-J) lymph nodes K) specimen 2 L) specimen 3.
  Note:
  1. The interpretation of MMR panel and recommended studies are as below: (1) No loss of nuclear expression of MMR proteins: low probability of microsatellite instability-high (MSI-H) . (2) Loss of nuclear expression of MLH1 and PMS2: testing for methylation of the MLH1 promoter and/or mutation of BRAF is indicated (the presence of a BRAF V600E mutation and/or MLH1 methylation suggests that the tumor is sporadic and germline evaluation is probably not indicated; absence of both MLH1 methylation and of BRAF V600E mutation suggests the possibility of Lynch syndrome, and sequencing and/or large deletion/duplication testing of germline MLH1 may be indicated). (3) Loss of nuclear expression of MSH2 and MSH6: high probability of Lynch syndrome (sequencing and/or large deletion/duplication testing of germline MSH2 may be indicated, and, if negative, sequencing and/or large deletion/duplication testing of germline MSH6 may be indicated). (4) Loss of nuclear expression of MSH6 only: high probability of Lynch syndrome (sequencing and/or large deletion/duplication testing of germline MSH6 may be indicated). (5) Loss of nuclear expression of PMS2 only: high probability of Lynch syndrome (sequencing and/or large deletion/duplication testing of germline PMS2 may be indicated). (6) Please note: There are exceptions to the above IHC interpretations. These results should not be considered in isolation, and clinical correlation with genetic counseling is recommended to assess the need for germline testing.
  2. This case has been peer reviewed by two doctors.
  #T-59300_2 #M-81403_2 2 1105 S0000F
  #T-C4560_2 #M-09183_2 0 1105 000000

其它

出院時情況

- 3.改門診治療

出院指示

藥名	劑量	劑量單位	用法	頻次	數量	數量單位	境況	天數	單位
Chlorzoxazone-250	1	TAB	PO	TID	42	TAB	TH	14	天
AVOdart cap 0.5mg	1	CAP	PO	QD	14	CAP	TH	14	天
Estazolam tab 2mg	1	TAB	PO	HSPRN	7	TAB	TH	7	天
500mg Glucophage	1	TAB	PO	BID	28	TAB	TH	14	天
60mg Diamicron MR	0.5	TAB	PO	QD	7	TAB	TH	14	天
10mg Crestor tab	1	TAB	PO	QPM	14	TAB	TH	14	天
Ultracet tab	1	TAB	PO	Q8HPRN	21	TAB	TH	7	天
Acetylcysteine tab	1	TAB	PO	QD	7	TAB	TH	7	天
50mg Betmiga tab	1	TAB	PO	HS	14	TAB	TH	14	天

出院計劃

主治醫師_2062F 蔣鋒帆 / 住院醫師_2331F 陳凱祥

正式摘要(電子簽章：000000205236888)

W52 / W52

doctorRank:B
